# The impact of biofeedback in enhancing chronic pain rehabilitation: A systematic review of mechanisms and outcomes

**DOI:** 10.1016/j.heliyon.2025.e41917

**Published:** 2025-01-11

**Authors:** Andrea Calderone, Vincenza Maura Mazzurco Masi, Rosaria De Luca, Antonio Gangemi, Mirjam Bonanno, Daniela Floridia, Francesco Corallo, Giovanni Morone, Angelo Quartarone, Maria Grazia Maggio, Rocco Salvatore Calabrò

**Affiliations:** a Department of Clinical and Experimental Medicine, University of Messina, Piazza Pugliatti, 1, 98122, Messina, Italy; b Università Degli Studi di Palermo, Piazza Marina, 61, 90133, Palermo, Italy; c IRCCS Centro Neurolesi Bonino Pulejo, S.S. 113 Via Palermo, C.da Casazza, 98124, Messina, Italy; d Department of Life, Health and Environmental Sciences, University of L'Aquila, 67100, L'Aquila, Italy; e San Raffaele Institute of Sulmona, 67039, Sulmona, Italy

**Keywords:** Biofeedback, Chronic pain, Neurorehabilitation, Pain management, Therapeutic interventions, Patient outcomes

## Abstract

**Background and objectives:**

Chronic pain (CP), affecting approximately 20 % of adults globally, imposes a profound burden on individuals and healthcare systems. This condition, characterized by persistent pain, muscle stiffness, and emotional distress, often results in a complex interplay of physical and psychological factors that exacerbate symptoms and hinder recovery. Biofeedback (BFB), a non-invasive intervention, offers a promising rehabilitation strategy by enabling individuals to monitor and self-regulate physiological responses, such as muscle tension, heart rate, and skin temperature. Through this process, BFB disrupts the vicious cycle of pain and stress, fostering relaxation, reducing muscle strain, and alleviating emotional distress. This systematic review aimed to examine the mechanisms underlying BFB's therapeutic effects in CP rehabilitation, specifically its ability to enhance self-regulation and promote relaxation to improve pain control. Furthermore, it aimed to evaluate the impact of BFB on key outcomes, including pain severity, functional capabilities, and quality of life, with the goal of guiding its integration into contemporary rehabilitation practices.

**Materials and Methods:**

Following PRISMA guidelines, a systematic search was conducted in PubMed, Web of Science, and Embase (2014–2024) to identify studies on BFB for CP. Inclusion criteria included original research involving BFB as a primary or secondary intervention for CP, with outcomes related to pain management and rehabilitation. This review is registered on Open OSF (X5HPB).

**Results:**

BFB has shown consistent efficacy as a complementary therapy in CP management, offering significant reductions in pain intensity and enhancements in quality of life across diverse CP conditions. Mechanistically, BFB facilitates improved self-regulation by training patients to modulate physiological responses, such as muscle tension and heart rate variability, leading to better pain control and stress reduction.

**Conclusions:**

BFB shows significant promise as a supplementary treatment for different CP disorders. The evidence that was examined shows that it is effective in improving how pain is perceived, increasing functional results, and boosting overall quality of life among a variety of patient groups.

## Highlights

• **Chronic Pain (CP)** affects 20 % of adults, causing persistent pain and emotional distress.
• Biofeedback (BFB) shows promise as a non-invasive method for CP management.
• Systematic review of BFB's benefits for headaches, **Low Back Pain**, and fibromyalgia.
• BFB improves pain regulation and quality of life in CP patients.
• Further research is needed to optimize BFB for specific pain syndromes.

## 1 Introduction

Pain is an intricate and varied phenomenon that can affect a person's overall well-being [[1], [2], [3]]. It is typically categorized into two groups: acute pain, caused by injury or illness, and chronic pain (CP), lasting longer than the usual healing period, typically over three months [4,5]. CP can appear in different ways, such as headaches, neck pain, fibromyalgia, lower back pain (LBP), coccydynia and pelvic pain, all of which create significant challenges for both individuals and healthcare systems [[6], [7], [8]]. Epidemiological research shows that CP conditions are very common [[9], [10], [11], [12]], with approximately 20 % of adults experiencing CP, of which a notable amount report pain in multiple areas [[13], [14], [15], [16]]. Symptoms associated with CP can vary widely and may include enduring pain, muscle stiffness, fatigue, and reduced physical capability [17,18]. These signs frequently happen together with emotional suffering like anxiety and depression, making treatment and recovery more challenging [19,20]. Rehabilitation is crucial in CP [21], and pain management [22] as it assists patients in recovering lost functional abilities and enhancing their quality of life [23].

### 1.1 Biofeedback: an innovative approach to chronic pain management

Among the innovative strategies for managing chronic pain, biofeedback (BFB) stands out as a promising, non-invasive approach that empowers individuals to regulate their physiological responses and alleviate symptoms effectively. By utilizing devices that deliver real-time feedback on metrics such as heart rate, blood pressure, and skin temperature, patients can learn to modulate their physiological reactions to stress and discomfort effectively [24,25]. This process involves placing sensors on the body to monitor specific functions, with the feedback typically displayed on a screen for intuitive interpretation. For instance, individuals experiencing CP can identify patterns of muscle tension and practice relaxation techniques to mitigate their symptoms [26,27]. BFB holds particular relevance for CP, where the interplay of physical strain and emotional stress often intensifies the condition [28]. Through self-regulation techniques, BFB disrupts this vicious cycle, fostering relaxation and alleviating muscle tension [29,30]. Moreover, BFB can be adapted to address various pain conditions [[31], [32], [33]], offering a versatile and patient-centered approach to treatment [34,35]. Table 1 outlines the range of BFB methods employed in pain management [[36], [37], [38]]. Aligned with contemporary rehabilitation paradigms, BFB encourages patients to take an active role in their recovery [[39], [40], [41]], promoting greater understanding and mastery of their physiological responses. This empowerment contributes to improved well-being and overall quality of life [[42], [43], [44], [45], [46]]. This brings us to the purpose and objectives of our systematic review, which seek to analyze the available evidence of BFB in treating a wide range of CP conditions, which includes headaches, neck pain, fibromyalgia, pelvic pain, coccydynia and LBP, in patients undergoing rehabilitation. This systematic review aimed to examine the mechanisms underlying BFB's therapeutic effects in CP rehabilitation, specifically its ability to enhance self-regulation and promote relaxation to improve pain control. Furthermore, it aimed to evaluate the impact of BFB on key outcomes, including pain severity, functional capabilities, and quality of life, with the goal of guiding its integration into contemporary rehabilitation practices.

**Table 1:** A summary of the biofeedback techniques.

Biofeedback Techniques	Description
Electromyography (EMG)	EMG is a diagnostic technique used to assess the health and functioning of muscles and the nerve cells that govern them (motor neurons). It accomplishes this by detecting electrical activity in skeletal muscles. Provides extensive information on muscle activation and neuromuscular health, assisting in the diagnosis of a variety of muscular and neurological problems. There are two types of EMG: surface EMG, which is non-invasive and involves placing electrodes on the skin over the muscle, and intramuscular EMG, which is invasive and involves inserting a needle electrode directly into the muscle. It can help to diagnose conditions like amyotrophic lateral sclerosis, myasthenia gravis, and muscular dystrophy, as well as evaluate nerve dysfunctions like carpal tunnel syndrome and peripheral neuropathy, and identify muscle inflammation (myositis) and muscle weakness
Thermal Biofeedback (TBF)	TBF is a method that helps people learn to manage involuntary physiological processes like skin temperature. It is widely used to relieve stress, cure chronic pain, and improve illnesses such as migraine headaches and Raynaud's syndrome. Small sensors are affixed to the skin, usually on the fingers or toes, to detect skin temperature. The sensors are linked to a biofeedback device, which shows the temperature values in real time. Skin temperature is affected by blood flow, which is regulated by the autonomic nervous system. Stress and worry can produce vasoconstriction (the narrowing of blood vessels), which results in a decreased skin temperature. In contrast, relaxing can produce vasodilation (the widening of blood vessels), which raises skin temperature
Electrodermal Biofeedback (GSR)	GSR biofeedback is a technique that measures the electrical conductance of the skin, which varies with its moisture level. Since sweat gland activity is controlled by the autonomic nervous system and increases with stress or arousal, GSR provides a useful measure of psychological or physiological arousal. By providing real-time feedback on skin conductance, it helps individuals learn to control their stress responses through effective relaxation techniques. This method is beneficial for a range of applications, from stress management to improving focus and treating anxiety-related disorders, contributing to overall psychological well-being
Neurofeedback (EEG)	EEG is a therapy approach that teaches self-regulation of brain function by displaying brain activity in real time, most often via electroencephalography. Its goal is to assist people in enhancing their brain function and managing a variety of neurological and psychological problems. It is built on the idea that brainwave patterns can be monitored and trained. Individuals can learn to control their brain activity by offering feedback on these patterns. Electrodes are applied to the scalp to monitor electrical activity in the brain. The signals from the electrodes are amplified and sent to a computer for processing. The program analyzes EEG data and gives real-time feedback, often in the form of visual or audio cues. Neurofeedback may alter pain perception and treatment, with research focusing on neuronal plasticity, pain modulation, and psychosocial aspects
Heart Rate Variability (HRV)	HRV biofeedback is a technique that teaches people how to enhance their HRV (a measure of the variation in time intervals between successive heartbeats) through regulated breathing and relaxation exercises, which improves autonomic function and stress resilience. This metric represents the autonomic nervous system's control of the heart, representing the balance between the sympathetic (fight or flight) and parasympathetic (rest and digest) branches. Individuals participate in biofeedback sessions, which generally include guided breathing exercises aimed at improving HRV. Participants are instructed to breathe slowly and steadily (usually 5–7 breaths per minute) to stimulate the parasympathetic nervous system and boost HRV. The biofeedback equipment provides real-time HRV parameters, which are frequently represented by graphs or visual signs. It assists people with chronic pain disorders in managing their pain by lowering stress and increasing autonomic function

∗Legend: Electromyography (EMG), Thermal Biofeedback (TBF), Electrodermal Biofeedback (GSR), Neurofeedback (EEG), Heart Rate Variability (HRV).

## 2 Materials and Methods

### 2.1 Search strategy

**Fig. 1:**
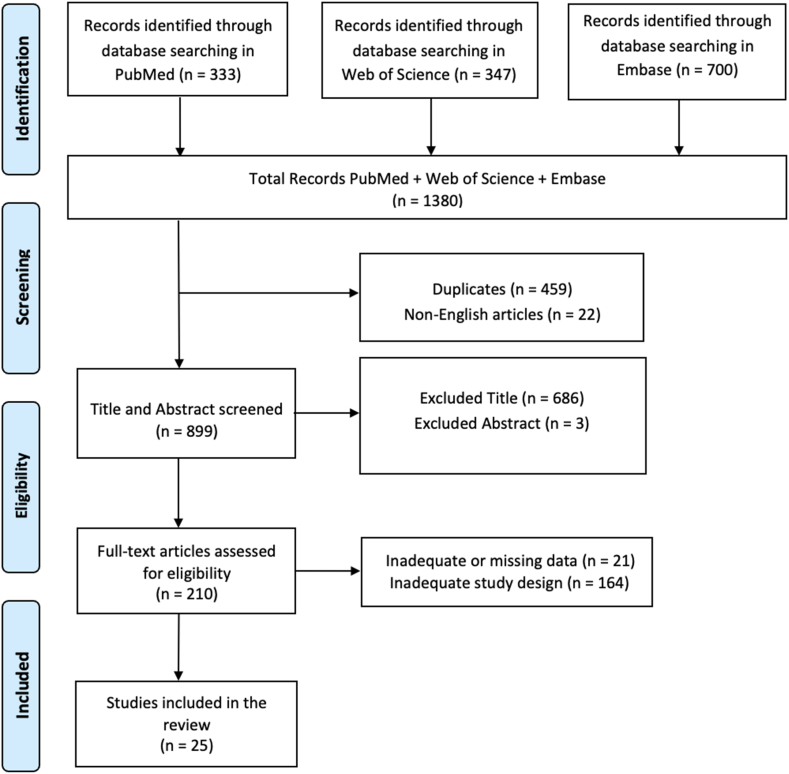
PRISMA 2020 flow diagram of evaluated studies.

This systematic review uses a methodical approach to evaluate research papers from 2014 to 2024. We conducted a comprehensive literature search (from April 15, 2024 to May 10, 2024) using PubMed, Web of Science, and Embase databases, employing the keywords (All Fields: “Biofeedback”) AND (All Fields: “Chronic Pain”). Searches were conducted independently by two reviewers (AC, MGM) using Boolean operators and controlled vocabulary (e.g., MeSH terms), to enhance transparency and accuracy in identifying relevant studies. The PRISMA (Preferred Reporting Items for Systematic Reviews and Meta-Analyses) flow diagram was utilized to outline the process (identification, screening, eligibility, and inclusion) for selecting relevant studies as illustrated in Fig. 1. The Cochrane Risk of Bias (RoB 2) tool was used to evaluate the risk of bias in randomized controlled studies, while the ROBINS-I tool was used for uncontrolled experimental papers in this review. Furthermore, all articles were screened based on titles, abstracts, and full texts by two researchers (AC, MGM), who independently performed data extraction, article collection, and cross-validation to reduce the other risk of bias (e.g., missing results bias, publication bias; time lag bias; language bias). Data items collected included study design, sample size, participant characteristics, type of pain condition, BFB intervention specifics, duration, outcomes measured, and results. Data synthesis was conducted using a narrative method, summarizing results from various studies due to the diversity in types of pain and BFB techniques. The researchers (AC and MGM) read full-text articles deemed eligible for the study, and in case of disagreement on inclusion and exclusion criteria, the final decision was made by a third researcher (RCS). Moreover, the agreement between the two reviewers (AC and MGM) was assessed using the kappa statistic. The kappa score was considered to indicate great agreement between the reviewers, with a substantial agreement threshold set at > 0.61. This standard guarantees a strong assessment of the consistency between raters, focusing on reaching a significant agreement in the process of data extraction. The articles were reviewed, filtered for relevance, and summarized to identify key topics based on inclusion/exclusion criteria. This review has been registered on OSF with the following DOI number: DOI 10.17605/OSF.IO/X5HPB.

### 2.2 PICO evaluation

We applied the PICO model (Population, Intervention, Comparison, Outcome) to create our search terms. The target population being studied in this systematic review consists of individuals who are suffering from CP. This will include conditions like headaches, neck pain, fibromyalgia, pelvic pain, coccydynia and LBP, characterized by their notable diversity. The intervention includes BFB techniques as a non-invasive approach to assist patients in pain management. We will analyze the outcomes of individuals who receive BFB and compare them to control groups who do not receive this treatment in order to determine the effects of BFB on reducing pain and overall rehabilitation progress.

### 2.3 Inclusion criteria

This systematic review will encompass research that investigates the application of BFB for managing CP disorders, including headaches, neck pain, fibromyalgia, pelvic pain, coccydynia, and LBP, in individuals receiving rehabilitation. Eligible studies should examine BFB as a primary or secondary treatment and provide details on pertinent pain-related outcomes, such as pain intensity, functional consequences, and quality of life. Only primary research will be taken into account, including randomized controlled trials, non-randomized experimental studies, cohort studies, and longitudinal studies. To maintain consistency, only studies released in English will be considered.

### 2.4 Exclusion criteria

Studies will be excluded if they do not concentrate on BFB as the main or secondary treatment for CP conditions or if they merge BFB with other unrelated therapies without isolating its particular effects. Studies that do not provide thorough explanations of the intervention techniques, processes, or results associated with BFB will be excluded. Furthermore, studies that do not provide information on pain-related outcomes or have inadequate data regarding the specified CP conditions will be excluded. Articles written in languages other than English, along with reviews (systematic, narrative, or integrative), will not be taken into account. Research that includes animal models or populations not related to CP conditions will also be omitted.

## 3 Results

### 3.1 Quality of included studies - risk of bias

**Fig. 2:**
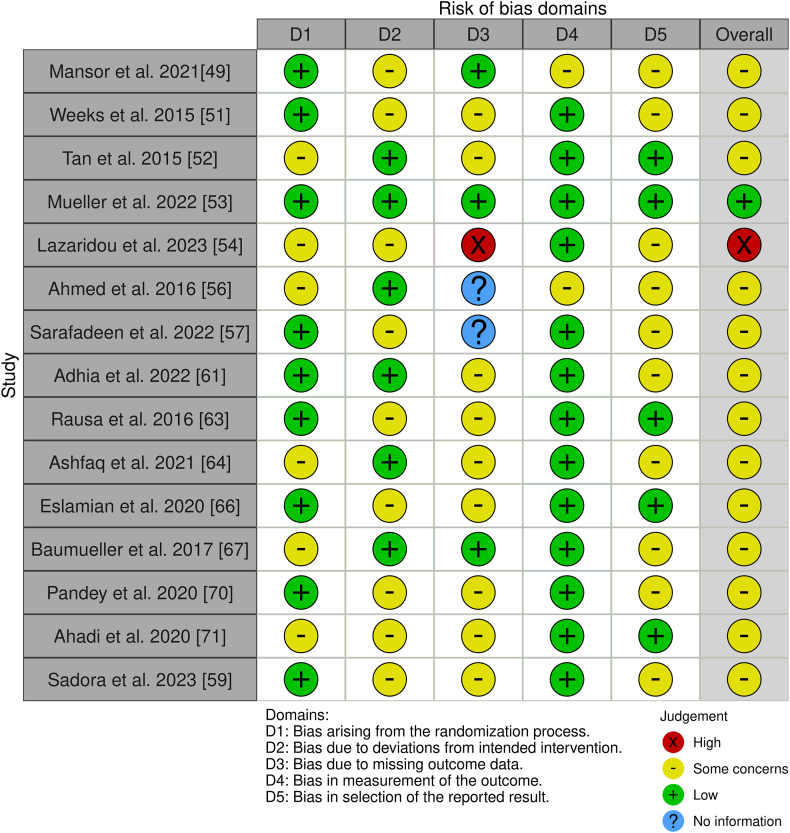
Risk of Bias (RoB) of included RCT studies.

We assessed the risk of bias using appropriate tools based on the design of the included studies [[47], [48], [49], [50], [51], [52], [53], [54], [55], [56], [57], [58], [59], [60], [61], [62], [63], [64], [65], [66], [67], [68], [69], [70], [71]]. Of the twenty-five studies, fifteen were a randomized controlled trial (RCT) [49,[51], [52], [53], [54],^56^,^57^,^59^,^61^,^63^,^64^,^66^,^67^,^70^,^71^]. For this one, we used the updated Cochrane Risk of Bias (RoB 2) tool, which covers five domains: i) bias arising from the randomization process, ii) bias due to deviations from the intended intervention, iii) bias due to missing data on the results, iv) bias in the measurement of the outcome and v) bias in the selection of the reported result (Fig. 2) [72].

**Fig. 3:**
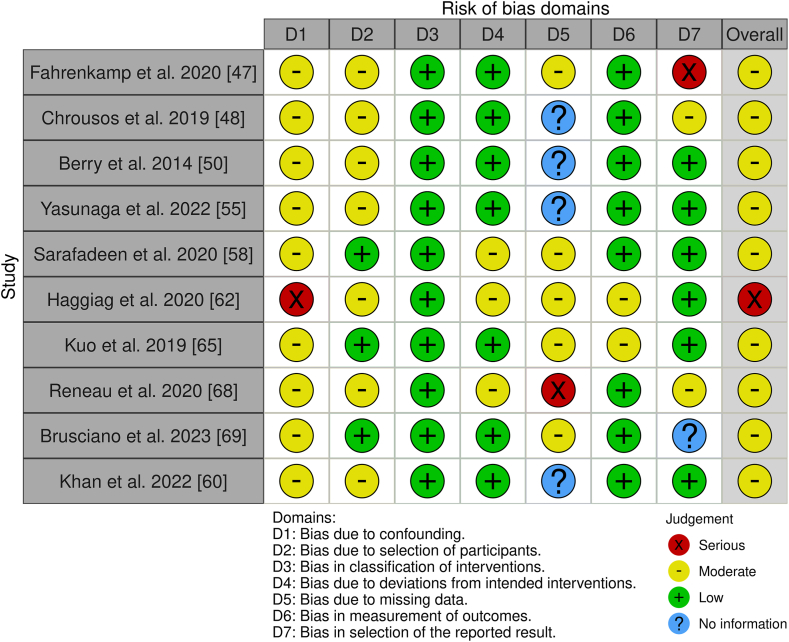
Cochrane risk of bias in non-randomized studies of interventions (ROBINS-I).

Our assessment of bias across the included studies revealed varying levels of methodological rigor, with some notable concerns affecting result reliability. Mansor et al. [49] and Weeks et al. [51] highlighted issues with the reporting of results (D5) and deviations from intended interventions (D2). Similarly, Tan et al. [52] identified shortcomings in the randomization process (D1) and missing outcome data (D3), which could compromise result interpretation. Lazaridou et al. [54] reported high risks in D1, D2, D3, and D5, underscoring concerns about outcome reliability. While Mueller et al. [53] demonstrated consistently low bias risks across all domains, Ahmed et al. [56] and Sadora et al. [59] flagged challenges related to missing data and intervention fidelity (D3 and D2). Overall, despite several studies showing low risks in critical areas, recurring issues with randomization, intervention adherence, and data completeness (D1, D2, D3) highlight the need for improved methodological rigor to enhance confidence in future findings. For the ten non-randomized studies – eight uncontrolled experimental studies [47,50,55,58,62,65,68,69], one multicenter controlled placebo study [48], and one cross sectional study [60] – we applied the ROBINS-I tool. ROBINS-I assesses bias in seven areas: i) bias due to confounding, ii) bias in participant selection, iii) bias in classification of interventions, iv) bias due to deviations from intended interventions, v) bias due to missing data, vi) bias in outcome measurement, and vii) bias in selection of the reported outcome (Fig. 3) [73].

The ROBINS-I evaluations reveal that the studies generally demonstrate moderate methodological quality, with varying risks across key domains. Significant bias in outcome selection (D7) was observed in Fahrenkamp et al. [47], raising concerns about result transparency, while Haggiag et al. [62] and Chrousos et al. [48] showed moderate risks across several domains. Confounding (D1) consistently posed a moderate risk, reflecting inadequate control of confounding variables. Participant selection (D2) also exhibited moderate risk in multiple studies, suggesting potential sampling biases. However, intervention classification (D3) was robust across all studies, with minimal risk reported. Deviations from intended interventions (D4) showed mixed results, with moderate risks in some cases potentially affecting intervention fidelity. Missing data (D5) posed moderate risks in several studies, impacting result reliability, while outcome measurement (D6) was generally reliable, with only a few studies showing moderate risks. Overall, while the findings provide some reassurance, the presence of moderate to serious biases in critical areas highlights the need for improved methodological rigor to strengthen confidence in future research outcomes.

### 3.2 Synthesis of evidence

In total, 1380 articles were found: 459 articles were removed due to duplication after screening; 22 articles were excluded because it was not published in English; 689 articles were excluded based on title and abstract screening. Finally, 185 articles were removed based on screening for inadequate and untraceable study designs (Fig. 1). Twenty-five research articles met the inclusion criteria and were therefore included in the review. These studies are summarized in Table 2.

**Table 2:** Summary of studies included in the research.

Author	Aim	Study Design/Intervention	Treatment Period	Sample Size	Sample Characteristics	Outcomes Measures	Main Findings
Mansor et al., 2021 [49]	To study how effective BEST is in reducing pain scores and serum cortisol levels in patients with CNP, in comparison to a placebo intervention.	Randomized Controlled Trial.	January 1st, 2014 to June 30th, 2014	20 patients.	*Age*: Average age of 53.5 years, with a deviation of 13.8; ages range from 31 to 82 years *Gender*: 8 males and 12 females.	VAS and serum cortisol levels assessed via direct chemiluminescence.	There was a significant decrease in pain scores in the BEST group, while the placebo group showed little to no change. The BEST group experienced a notable decrease in serum cortisol levels, whereas the placebo group did not exhibit a significant shift.
Weeks et al., 2015 [51]	To evaluate the effectiveness of HRV-BFB training in managing chronic pain among adults.	Randomized Controlled Trial.	Participants underwent BFB training for a duration of three weeks, consisting of a total of nine sessions.	20 adults.	*Age*: 18 years and older. *Gender*: not Specificated.	VAS, PDQ, TSK-11.	Members of the faded feedback group saw a decrease in current and worst pain levels at the end of training and follow-up, whereas the full feedback group did not exhibit any notable changes in pain levels. Furthermore, a greater number of participants in the group that received faded feedback continued practicing BFB skills after training, as opposed to the group that received full feedback.
Tan et al., 2015 [52]	To evaluate the effectiveness of self-hypnosis training and BFB relaxation techniques in treating CLBP	Randomized Controlled Trial.	8 weeks.	100 participants.	*Age*: Average age of 55 years. *Gender*: 79 % male; racial/ethnic distribution included Caucasian (47 %), African-American (32 %), Hispanic (15 %), and others (6 %).	BPI and PSQI.	Significant improvements in pain intensity, pain interference, and sleep quality were observed in both the hypnosis and BFB groups. In addition, individuals in the hypnosis groups were more inclined to report significant decreases in pain intensity that were considered clinically meaningful, as opposed to the BFB group.
Mueller et al., 2022 [53]	To explore how helpful game-based real-time BFB training is for individuals with chronic non-specific low back pain.	Randomized Controlled Trial.	Every participant went through a measurement session that lasted approximately 2 h.	13 participants.	*Age*: between 18 and 70 years. *Gender*: eight females and five males.	Highest angle achieved during side bending motion and personal normalized angle reproduction.	The research discovered that there was no notable decrease in CLBP according to the VAS assessments pre and post intervention. Furthermore, no negative impacts were documented throughout the investigation.
Lazaridou et al., 2023 [54]	To assess how helpful EMG-BFB is as a treatment for individuals with CLBP.	Randomized Controlled Trial.	8 weeks.	81 individuals were enlisted; 50 successfully finished the research.	*Age*: range of 18–65 years. *Gender*: primarily female participants.	Self-report surveys cover demographics, pain intensity (main focus), pain disruption, adverse emotions, impairment (additional measures), and QST.	The EMG-BFB group exhibited notable enhancements in pain intensity in contrast to the control group, with secondary results revealing shifts in pain interference and disability over time with no specific effects based on group.
Ahmed et al., 2016 [56]	To assess if adding EMG-BFB to trunk stabilization exercises improves outcomes in CLBP patients compared to just doing trunk stabilization exercises.	Randomized Controlled Trial.	Interventions were carried out twice a week over a period of 8 weeks.	90 patients.	*Age*: 20–40 years. *Gender*: all males.	The main result was the TrA activation ability, assessed with a pressure BFB unit; other results were pain level (measured by VAS) and functional impairment (measured by MODQ).	The research showed notable enhancements in TrA activation ability, pain level, and functional impairment in the EMG-BFB group compared to the control group, especially evident at the 6 and 8-week points. In general, performing trunk stabilization exercises along with EMG-BFB resulted in superior results compared to exercises that did not include BFB.
Sarafadeen et al., 2022 [57]	To assess how well LSE with RUSI-BFB works for treating LMM CSA, pain, functional disability, and quality of life in people with non-specific CLBP.	Randomized Controlled Trial.	8 weeks, with interventions given two times weekly.	A combined 90 individuals, with an equal distribution of 30 in each of the three intervention groups.	*Age*: between 18 and 60 years. *Gender*: both male and female.	The main result is the measurement of muscle cross-sectional area using RUSI, while additional outcomes consist of self-reported questionnaires assessing pain, functional disability, and quality of life.	Subjects who were given LSE along with RUSI BFB showed significant enhancements in LMM CSA, decreased pain, lower functional disability, and improved quality of life in comparison to those who were given standard LSE or minimal intervention.
Adhia et al., 2022 [61]	To investigate if a new EEG neurofeedback technique that targets specific brain regions involved in pain processing can be used to treat CLBP effectively and safely.	Randomized Controlled Trial.	4 weeks with 3 sessions per week.	60 participants.	*Age*: between 18 and 75 years. *Gender*: both genders included.	BPI, NRS, RDQ.	The neurofeedback therapy was proven to impact the way pain is processed and boost mechanisms that prevent pain in individuals, resulting in enhancements in their self-reported pain levels and abilities. Individuals who were given the real treatment showed more positive clinical results in contrast to those in the fake treatment group.
Rausa et al., 2016 [63]	To assess how well EMG-BFB works for patients suffering from chronic migraines and medication overuse headaches.	Randomized Controlled Trial.	1 month.	27 participants.	*Age*: not Specificated. *Gender*: not Specificated.	PRSS, PRCS and headache diary.	The BFB group showed a notable decrease in both how often they experienced headaches and how much pain medication they needed, when compared to the control group. Additionally, more patients in the BFB group returned to having occasional headaches by the end of the treatment and follow-up evaluations.
Ashfaq et al., 2021 [64]	Assessing the efficacy of craniocervical flexion training in individuals suffering from chronic neck pain, both with and without pressure BFB.	Randomized Controlled Trial.	Between May 2019 and December 2019.	30 participants.	*Age*: 25–40 years *Gender*: 20 females and 10 males.	NPRS, DNF, CCFT.	Craniocervical flexion training using pressure BFB resulted in notable enhancements in both pain and endurance ratings when compared to the control group. Both interventions led to significant improvements in the evaluated criteria throughout the duration of the study.
Eslamian et al., 2020 [66]	Assessing how well acupuncture decreases neck pain and dysfunction in MPS patients compared to BFB therapy.	Randomized Controlled Trial.	March 2018–2019.	50 patients.	*Age*: 25–55 years. *Gender*: both genders included.	NDI, VAS, cervical spine ROM, PPT.	Acupuncture and BFB therapy both demonstrated effectiveness in decreasing neck pain and dysfunction, leading to significant clinical improvements in outcomes throughout the treatment duration.
Baumueller et al., 2017 [67]	To assess the efficiency of EMG-BFB training in women diagnosed with fibromyalgia.	Randomized Controlled Trial.	8 weeks with a total of 14 EMG-BFB sessions.	40 patients.	*Age*: 18–65 years. *Gender*: exclusively female.	FIQ, SF-36, PGIC, BDI and SCL-90-R.	By the end of the intervention, both the treatment and control groups saw enhancements in FIQ scores and secondary outcome measures. Nevertheless, there were no notable distinctions discovered among the groups.
Pandey et al., 2020 [70]	To assess the effectiveness of conventional treatment compared to BFB and pelvic-floor muscle relaxation therapy in patients with chronic pelvic pain/chronic pelvic pain syndrome who did not respond to medication.	Randomized Controlled Trial.	12 weeks of treatment, with a 3-month check-up afterwards.	84 patients.	*Age*: 18 and 40 years. *Gender*: both male and female.	NIH-CPSI.	Following 3 months of therapy, both sets of participants showed notable enhancements in NIH-CPSI scores; yet, a greater proportion of individuals in the BFB group sustained their progress during the 3-month evaluation compared to those in the traditional treatment group.
Ahadi et al., 2020 [71]	To evaluate how BFB therapy along with pelvic floor muscle exercises impacts pain and quality of life in female patients with chronic coccydynia.	Randomized Controlled Trial.	From May 2015 to May 2016, follow-up evaluations were conducted 4, 8, and 24 weeks after starting treatment.	A total of 30 patients were divided into two groups, with 15 patients in each group.	*Age*: The BFB group had an average age of 41.4 years (SD = 8.96), while the exercise group had an average age of 35.6 years (SD = 10.8). *Gender*: All females.	VAS, DPQ, SF-36.	Both groups saw notable enhancement in average VAS pain ratings at 1, 2, and 6 months after receiving treatment. There was no notable variance among the groups in reductions of VAS pain scores as time progressed. Improvements were seen within the groups in the DPQ and SF-36 Quality of Life scores, especially in physical functioning and overall quality of life for both groups, with different domains showing enhancements in each group.
Sadora et al., 2023 [59]	To investigate how sEMG-BFB affects CLBP and its influence on physical function, sleep, pain catastrophizing, anxiety, and depression.	Randomized Controlled Trial.	8 weeks.	26 participants.	*Age*: average of 45 years. *Gender*: primarily women who were White and not Hispanic.	BPI, ODI, HADS.	Individuals noted considerable enhancements in pain control and general state of health. The benefits of virtual sessions and heightened awareness of pain management strategies were highlighted.
Chrousos et al., 2019 [48]	The research aimed to assess how effective a non-invasive electrodermal BFB device (RegMatEx) is in managing perceived pain and chronic systemic inflammation in patients with chronic pain and MUS.	Multicenter Placebo Controlled Study.	During a period of more than three weeks, each participant will undergo six sessions of electrodermal BFB lasting 30 min each, conducted twice a week.	The research involved a group receiving treatment consisting of 1015 individuals and a group receiving a placebo consisting of 950 individuals.	*Age*: between 30 and 86 years. *Gender*: In the treatment group, there were 401 males and 614 females, with an average age of 48 years. There were 500 men and 450 women in the control group, and their average age was 50 years old.	NRS, CRP.	There was a significant decrease in perceived pain on the NRS scale and a reduction in CRP concentrations in the treatment group. There were no notable differences in pain perception or CRP levels among the participants in the placebo group.
Fahrenkamp et al., 2020 [47]	To assess the results of BFB treatments in pediatric pain rehabilitation for teenagers with persistent pain.	Uncontrolled experimental Study.	17 days.	104 participants.	*Age*: 12–18 years. *Gender*: mixed gender.	RR, sEMG	Participants demonstrated notable enhancements in their ability to regulate physiological functions, such as reducing muscle tension and respiratory rates, while also exhibiting increased confidence in implementing relaxation techniques in their everyday tasks.
Berry et al., 2014 [50]	To assess the impact of using a self-regulation method along with computerized HRV-BFB on pain, stress, and emotional health in veterans suffering from chronic pain.	Uncontrolled experimental Study.	4 weeks.	14 veterans.	*Age*: not Specificated. *Gender*: not Specificated.	BPI, PSS, HRV assessments.	The treatment group showed notable enhancements in HRV coherence, along with substantial decreases in pain ratings, perceived stress, negative emotions, and limitations in physical activity. These changes were significantly more noticeable than the ones seen in the control group.
Yasunaga et al., 2022 [55]	Assessing the efficiency of outpatient BFB physical therapy with the HAL lumbar type for individuals suffering from CLBP.	Uncontrolled experimental Study.	Not Specificated.	35 participants.	*Age*: mean age at 58 years with a range from 20 to 83. *Gender*: 14 men and 21 women.	VAS, FFD, SLR, and Thomas test.	After receiving BFB therapy using the HAL lumbar device, individuals experienced notable enhancements in their low back pain and hip flexibility. This was shown through significant improvements in multiple evaluated measures, demonstrating the therapy's effectiveness. There were no negative incidents mentioned throughout the examination.
Sarafadeen et al., 2020 [58]	To assess how a stabilization workout plan using ultrasound BFB impacts the lumbar multifidus muscle size, pain levels, functional limitations, and overall quality of life in individuals suffering from long-term lower back pain.	Uncontrolled experimental study.	6 weeks.	12 participants were enrolled with 10 completing the intervention.	*Age*: 18–60 years. *Gender*: both male and female.	lumbar multifidus muscle CSA.	The treatment resulted in notable enhancements in lumbar multifidus muscle CSA, pain severity, and functional disability, demonstrating beneficial impacts on these medical results, with no noticeable alterations seen in quality of life.
Haggiag et al., 2020 [62]	To assess how well an intraoral device (DIVA®) reduces orofacial pain and migraine symptoms in patients with awake bruxism.	Uncontrolled Experimental Study.	90 days.	74 patients.	*Age*: mean age of 38.8 years. *Gender*: 75.7 % female and 24.3 % male.	PIPS and VAS.	After 30 days of using the intraoral device, there were noticeable decreases in pain levels. These improvements continued even after stopping the device, showing that self-management of awake bruxism and pain symptoms was effective.
Kuo et al., 2019 [65]	To examine the instant impacts of live postural BFB on spinal alignment, muscle usage, and self-reported neck and shoulder discomfort while working on a computer.	Uncontrolled Experimental Study.	The entire test lasted more than 2 h, including two typing activities that each went on for 1 h.	21 adults.	*Age*: mean age of 23.8 *Gender*: 13 women and 8 men.	NRS and NDI.	Participants showed better spinal posture and reduced neck flexion and thoracic angles when utilizing BFB, resulting in a notable decrease in muscle activity of the cervical erector spinae. Self-reported neck and shoulder pain significantly rose after the typing tasks, regardless of the feedback given.
Reneau et al., 2020 [68]	To determine if the HRV-BFB protocol is possible and well-received by Veterans with fibromyalgia.	Uncontrolled Experimental Study.	Eight sessions, each lasting 1 h and held once a week.	10 participants.	*Age*: 33–68 years *Gender*: 7 women and 3 men.	CEQ, FIQR, SFMQ and HRC.	The research found that participants generally found the HRV-BFB intervention acceptable, with some slight improvements in functional status and quality of life noted, while pain levels stayed mostly the same.
Brusciano et al., 2023 [69]	To assess how well a rehabilitation protocol utilizing radiofrequency diathermy works in patients with anorectal functional pain syndrome and paradoxical pelvic floor contraction.	Uncontrolled Experimental Study.	Between September 2021 and May 2022.	30 patients.	*Age*: median age of 54 years. *Gender*: 66.6 % female and 33.3 % male.	VAS, CRAIQ-7 and HRAM.	Significant improvements in pain levels and quality of life, as well as a decrease in inaccurate muscle movements and an increase in diaphragmatic breathing were observed in patients after treatment.
Khan et al., 2022 [60]	To assess the consistency among different raters of the PBU in abdominal drawing-in tests for individuals with CLBP and healthy participants.	Cross sectional Study.	Between February 2021 to March 2021.	16 participants.	*Age*: 26–28 years. *Gender*: The study involved 8 people suffering from CLBP, with an equal gender distribution, and 8 healthy participants, with a higher percentage of females.	PBU was used for assessing muscle activation in abdominal drawing-in.	The research showed strong consistency among raters in assessing muscle activation using the PBU in both CLBP patients and healthy individuals, confirming its effectiveness.

Legend: Biofeedback (BFB), Visual Analog Scale (VAS), Dallas Pain Questionnaire (DPQ), Short Form (36) Health Survey (SF-36), medically unexplained symptoms (MUS), Numeric Rating Scale (NRS), C-reactive protein (CRP), Bio-Electrotherapy Stimulation Technology (BEST), chronic neuropathic pain (CNP), Heart Rate Variability (HRV), Pain Disability Questionnaire (PDQ), Tampa Scale of Kinesiophobia (TSK-11), chronic low back pain (CLBP), Brief Pain Inventory (BPI), Pittsburgh Sleep Quality Index (PSQI), Electromyography (EMG), Quantitative Sensory Testing (QST), transverse abdominis (TrA), Modified Oswestry Disability Questionnaire (MODQ), lumbar stabilization exercises (LSE), eal-time ultrasound imaging (RUSI), lumbar multifidus muscle cross-sectional area (LMM CSA), Electroencephalography (EEG), Brief Pain Inventory (BPI), Roland-Morris Disability Questionnaire (RDQ), Pain Related Self Statements Scale (PRSS), Pain Related Control Scale (PRCS), Deep Neck Flexor (DNF), Craniocervical Flexion Test (CCFT), myofascial pain syndrome (MPS), Neck Disability Index (NDI), range of motion (ROM), Pressure Pain Threshold (PPT), Fibromyalgia Impact Questionnaire (FIQ), Patient's Global Impression of Change (PGIC), Beck Depression Inventory (BDI), Symptom Checklist 90 Revised (SCL-90-R), National Institutes of Health Chronic Prostatitis Symptom Index (NIH-CPSI), Respiratory rate (RR), surface electromyography (sEMG), Perceived Stress Scale (PSS), Hybrid Assistive Limb (HAL), finger-to-floor distance (FFD), straight leg raising test (SLR), cross-sectional area (CSA), Oswestry Disability Index (ODI), Hospital Anxiety and Depression Scale (HADS), Percentage Improvement in Pain Scale (PIPS), Credibility/Expectancy Questionnaire (CEQ), Revised Fibromyalgia Impact Questionnaire (FIQR), Short-form McGill Pain Questionnaire (SFMQ), and Heart Rate Variability coherence (HRC), Colorectal and Anal Impact Questionnaire (CRAIQ-7), high-resolution anorectal manometry (HRAM), Pressure Biofeedback Unit (PBU).

Five research studies examined how BFB affects the way people experience pain, specifically looking at long-lasting pain conditions [[47], [48], [49], [50], [51]]. Also, ten studies looked into how effective BFB is in helping with LBP rehabilitation [[52], [53], [54], [55], [56], [57], [58], [59], [60], [61]], while another five studies explored the use of BFB in reducing headaches and neck pain [[62], [63], [64], [65], [66]]. Finally, five articles investigated the advantages of BFB in fibromyalgia and pelvic pain [[67], [68], [69], [70], [71]]. A qualitative analysis was conducted to synthesize findings from the studies, uncovering common themes and trends in the use of BFB for managing CP. Through thematic synthesis, we categorized studies based on their key outcomes, highlighting similarities and differences in approaches, treatment settings, and patient responses. This method also accounted for the certainty of evidence, offering a clearer perspective on the reliability of the results. The synthesis provides a comprehensive narrative on BFB's effectiveness across various CP conditions, deepening our understanding of its therapeutic potential.

### 3.3 Impact of biofeedback on pain perception: insights from chronic pain studies

BFB has emerged as a promising treatment for CP, helping patients improve pain self-regulation. Five studies were reviewed, encompassing various demographics, from adolescents to veterans. A study with 104 teenagers in CP rehabilitation found that BFB led to reduced muscle tension and improved pain management during daily activities [47]. Another trial of 2065 adults using electrodermal BFB showed decreased pain and inflammation in the treatment group, enhancing quality of life [48]. Another research study involving 20 neuropathic pain patients found that BFB electrostimulation therapy resulted in significant pain relief and hormonal responses [49]. In veterans, heart rate variability (HRV) BFB significantly reduced pain and stress [50]. A randomized controlled trial using HRV BFB with 20 adults demonstrated that reduced feedback schedules resulted in more sustained pain relief and better retention of BFB skills [51]. Overall, while the evidence varies in strength, these studies support BFB as an effective supplementary therapy for CP, especially with larger, multicenter trials needed to confirm its broader applicability. On the whole, the findings suggest a steady pattern of better pain control for a range of CP problems. The level of certainty in the evidence differs, with a comprehensive multicenter study offering strong data backing the success of electrodermal BFB, whereas smaller studies, although positive, require more research to validate effectiveness and applicability.

Ultimately, these studies provide compelling evidence supporting the effectiveness of BFB as a helpful additional therapy for CP, underscoring the importance of conducting larger randomized controlled trials to enhance the existing evidence.

### 3.4 The effectiveness of biofeedback in chronic low back pain rehabilitation

LBP poses a major health issue that often requires complex treatment approaches. This paragraph summarizes existing studies on how BFB interventions can enhance functional outcomes and decrease pain levels in individuals with LBP. A randomized controlled trial included 100 veterans with LBP, discovering that just two sessions of self-hypnosis with audio recordings might be as effective as eight sessions (in comparison to BFB), highlighting the need to investigate the optimal frequency and duration of practice at home. The evidence's certainty was moderate because of the sample size and its applicability to other groups [52]. Mueller and colleagues [53] involved 13 patients utilizing game-driven real-time feedback on trunk movement and observed enhanced control over movement, although changes in lateral flexion were minimal [53]. In another research, a group of 90 patients participated in an 8-week virtual electromyography (EMG) BFB program. Findings indicated a notable decrease in pain severity and enhancements in pain impact and limitation, as well as raised thresholds for low back pain [54]. An uncontrolled experimental study assessed 35 participants in a single BFB therapy session utilizing a hybrid assistive limb (HAL). The results showed notable improvements in hip flexibility and pain levels while moving. There were no negative side effects reported, suggesting that HAL BFB could provide immediate advantages in managing pain [55]. Another randomized controlled trial assessed 90 individuals participating in core strengthening workouts, with half of them using EMG-BFB. Both groups demonstrated significant enhancements in transverse abdominis activation ability, pain levels, and functional impairment [56]. Another randomized controlled trial included 90 individuals with non-specific LBP who were divided into three groups: lumbar stabilization exercises (LSE), LSE with real-time ultrasound imaging (RUSI) BFB, and minimal intervention. Findings showed that individuals who received LSE with RUSI saw a notable rise in the lumbar multifidus muscle's size and considerable decreases in pain and disability within eight weeks, showing the effectiveness of RUSI-BFB in rehabilitation [57]. Another research was conducted with a group of 10 individuals, who underwent spinal stabilization exercises with RUSI-BFB twice a week for a period of six weeks. The findings indicated notable improvements in the lumbar multifidus muscle size (P < 0.05, d = 1.03), pain (P < 0.001, d = 2.56), and disability (P < 0.05, d = 1.43) [58]. During the COVID-19 pandemic, virtual EMG-BFB therapy showed decreased pain and stress, better sleep, and increased self-awareness in 26 participants over an eight-week period [59]. A cross-sectional study, which included 16 participants with and without LBP, evaluated how dependable a pressure BFB unit is for measuring abdominal muscle activity. The results showed great consistency between raters [60]. Finally, a randomized trial involving infraslow neurofeedback with 60 patients found pain relief and enhanced brain connectivity, showing changes that were negatively correlated with pain severity during follow-up [61]. The effectiveness of BFB techniques like RUSI and surface EMG in enhancing muscle activation, alleviating pain, and improving the quality of life in LBP patients is underscored by all these studies, with certainty of evidence varying from high to moderate certainty.

### 3.5 Examining the role of biofeedback in alleviating headache and neck pain

Chronic headaches and neck discomfort are frequent problems that greatly impact overall well-being. This review explores the possibilities of BFB as a non-invasive therapy for these issues. Research involving 74 participants indicated that BFB utilizing a posterior interocclusal device markedly alleviated pain in the headache and neck areas, with effects lasting up to 360 days [62]. A study involving 27 patients indicated that frontal EMG-BFB, when paired with medication, lowered headache frequency and medication usage, yielding superior outcomes in the BFB group after four months [63]. In research involving 30 participants suffering from chronic neck pain, the combination of BFB and craniocervical flexion training enhanced muscle endurance, with advantages maintained for six weeks [64]. Research involving 21 adults indicated that using a postural BFB device enhanced spinal alignment and diminished muscle tension while using a computer for extended periods, providing temporary pain relief [65]. A study involving 50 patients examined BFB and electroacupuncture for cervical myofascial pain syndrome, determining that both treatments were effective, with electroacupuncture yielding superior outcomes [66]. In general, BFB demonstrates potential for alleviating headache and neck discomfort, boosting muscle endurance, and improving posture. Nevertheless, the caliber of the evidence differs, with certain constraints impacting the conclusions.

### 3.6 The benefits of biofeedback in fibromyalgia, pelvic pain and coccydynia

Fibromyalgia and pelvic pain pose considerable treatment difficulties, yet BFB provides encouraging advantages in managing symptoms. A randomized study involving 36 fibromyalgia patients over 8 weeks revealed that EMG-BFB raised pressure-pain thresholds in the trapezius muscles, although it did not enhance overall health status as evaluated by the Fibromyalgia Impact Questionnaire [67]. In a comparable 7-week investigation involving 10 veterans utilizing HRV-BFB, there were no notable changes in pain ratings; however, it noted enhancements in functional status and quality of life, indicated by an 18.1-point rise in Fibromyalgia Impact Questionnaire Revised scores [68]. In research involving 30 patients with anorectal functional pain syndrome, BFB alongside radiofrequency diathermy decreased pelvic floor contractions and enhanced pain relief and quality of life. Although enhancements were backed by objective assessments such as anorectal manometry, the lack of randomization and a control group constrained the robustness of causal conclusions, leading to moderate certainty of evidence [69]. A study with 84 men suffering from chronic prostatitis/pelvic pain syndrome revealed that pelvic-floor BFB maintained symptom relief at 6 months, in contrast to standard treatment. The random design and prolonged follow-up offered robust evidence, although more varied samples are required to verify external validity [70]. On the other hand, research involving 30 women suffering from coccydynia indicated that there was no extra advantage from BFB when used alongside pelvic floor exercises and corticosteroid injections, backed by low certainty owing to the small sample size and minimal differential effects observed between groups [71]. In summary, BFB demonstrates promise in reducing pain and enhancing function in certain conditions; however, differences in results and study design limitations emphasize the necessity for more thorough research.

## 4 Discussion

The results of this systematic review indicate that BFB is a promising adjunctive therapy for CP, providing potential advantages across various conditions, such as LBP, fibromyalgia, headaches, and pelvic pain. These findings highlight BFB's ability to enable patients to handle pain by enhancing self-regulation and control over their physiological reactions.

### 4.1 Integrating biofeedback into multimodal pain therapies and clinical outcomes

BFB interventions work by enhancing patients' awareness and regulation of physiological processes such as muscle tension, HRV, and electrodermal activity. This mechanism underlies the observed improvements in pain intensity, functional outcomes, and quality of life across different CP conditions [[74], [75], [76], [77]]. When used alongside additional treatments like physical therapy, cognitive-behavioral therapy, or medication, BFB can enhance the overall success of therapy plans [[78], [79], [80]]. For example, in fibromyalgia, BFB showed potential in increasing pressure-pain thresholds, though its impact on overall health status was limited. Similarly, BFB demonstrated its utility in enhancing functional capacity and reducing pain in LBP, with approaches such as EMG and ultrasound-based BFB proving particularly effective in activating core muscles and reducing disability [81,82]. The efficacy of BFB appears to be influenced by the specific CP condition and individual patient characteristics. Furthermore, research indicates that BFB may assist individuals in improving their coping skills, decreasing pain-related anxiety, and boosting their awareness of pelvic floor muscles for relaxation [83,84]. This is especially advantageous for people with pelvic pain, as they frequently encounter intricate pain that can be impacted by both physical and psychological elements. Likewise, BFB has shown encouraging outcomes for patients experiencing headaches and neck pain. The proofs suggests that BFB can result in decreases in how often headaches occur and how severe they are, along with enhancements in cervical function overall [85,86]. However, variability in outcomes was noted; for instance, BFB interventions targeting pelvic pain showed substantial benefits in pain reduction and pelvic floor relaxation, yet results were inconsistent in coccydynia, where its additive value to standard treatments was limited.

### 4.2 Biofeedback mechanisms

*Bridging Neuroplasticity and Pain Rel*ief BFB goes beyond enhancing physiological awareness and regulation; it actively promotes neuroplastic changes via persistent practice and feedback, possibly changing maladaptive neural circuits linked to CP. By providing real-time feedback, BFB allows patients to retrain motor and autonomic responses, fostering the establishment of healthy physiological patterns. For example, EMG-based BFB has been shown to be beneficial in motor re-education, focusing on specific muscle groups to enhance motor control and diminish compensatory processes that are typically associated with CP syndrome [54,56,59]. Furthermore, BFB has a significant effect on central sensitization, a major aspect of CP that is defined by increased central nervous system sensitivity and exaggerated pain responses. BFB may assist to relieve these exaggerated reactions by modulating autonomic nervous system activity and decreasing hypervigilance to pain stimuli. It also helps to improve interoceptive awareness, which allows people to perceive and interpret internal body signals more accurately. This improvement not only helps with pain control but also with emotional regulation, highlighting the many advantages of BFB in CP therapy.

### 4.3 Challenges and opportunities for optimization

Despite positive trends, significant heterogeneity exists in study methodologies, sample sizes, and intervention protocols, affecting the overall strength of evidence. Randomized controlled trials with larger and more diverse populations are essential to standardize and validate the most effective BFB approaches for specific CP conditions. Additionally, understanding patient-specific factors, such as psychological and behavioral characteristics, that influence BFB outcomes will be critical in optimizing treatment protocols.

### 4.4 Clinical advancements in rehabilitation

Various clinical progress needs to be considered. A major development that emerges from the studies reviewed is the incorporation of wearable technology, enabling patients to participate in self-monitoring and receive real-time feedback while going about their daily routines [87,88]. This empowerment encourages patients to be more involved in their rehabilitation, potentially resulting in better adherence to treatment plans. Additionally, progress in neurofeedback, is being investigated for its ability to improve cognitive and emotional functions during rehabilitation and in the treatment of pain [89]. This application highlights the importance of treating rehabilitation in a holistic manner, taking into account both physical and psychological aspects of well-being. Another significant clinical use is blending BFB with cognitive-behavioral therapies. Combining these methods can improve pain management techniques as patients become skilled at identifying and changing unhelpful thoughts linked to pain. This joint effort helps improve patient results and strengthen their ability to cope through a more thorough management plan. In addition, BFB is being integrated with virtual reality environments to produce immersive healing experiences. These new environments enable patients to test relaxation methods and pain control strategies in supervised settings, which can be especially beneficial for conditions such as fibromyalgia and chronic headaches. The combination of virtual reality and BFB offers a special chance for learning new skills in an exciting and interactive way [90]. In the realm of treating LBP, there is growing proof that BFB techniques, such as RUSI, can be effective in directing core stabilization exercises. This technique not only enhances muscle activation but also offers prompt feedback, which can boost patient motivation and result in improved long-term results. Moreover, the use of telehealth platforms has broadened the availability of BFB interventions, enabling patients to interact with practitioners from a distance [91]. This level of accessibility is essential for offering continuous assistance and direction, especially for individuals facing mobility obstacles or residing in isolated regions. Telehealth methods also allow healthcare providers to constantly track patient improvement and make necessary adjustments to treatment plans in a timely manner [92]. Ongoing research and advancements in BFB methods are expected to confirm its importance in rehabilitation practices.

### 4.5 Strengths and limitations

The systematic review provides a fair assessment of the pros and cons related to incorporating BFB into CP management. One of its major strengths lies in the extensive inclusion of studies, with a total of 25 articles reviewed, and its rigorous methodology following established guidelines like PRISMA. Using a thorough search strategy in various databases guarantees a diverse selection of studies were included, ultimately improving the credibility of the results. The review covers a wide range of evidence showcasing the effectiveness of BFB as a treatment by including a diverse group of people with different CP issues. A different important aspect is the implementation of the PICO model for distinct identification of the population, intervention, comparison, and outcomes. This organized method helps in systematically assessing how effective BFB is for various CP conditions, giving a better understanding of its possible advantages. The multifaceted limitations of this systematic review should be carefully taken into account. Although an extensive search strategy was used on significant databases, the study only included research from 2014 to 2024, possibly missing out on important studies before that period. Moreover, limiting the studies to only those in English could have created a language bias that restricted the applicability of the results. The diversity of pain conditions and BFB interventions being researched presents an additional challenge; the differences in research methods, results analysis, and patient groups make it difficult to combine findings and reach firm conclusions. Additionally, the assessment used narrative synthesis because of the variety of BFB methods and types of pain, which could restrict the ability to quantitatively evaluate the effectiveness overall. Furthermore, certain studies analyzed also had limited sample sizes, leading to doubts about the statistical strength and dependability of their results. Using bibliographies from different systematic reviews to find studies is another constraint, as it could affect the repeatability, which is crucial, and may also lead to bias. Relying on self-reported outcomes in various studies could also lead to bias since individuals' subjective views on pain can differ greatly. Hence, although the review offers important insights on the efficacy of BFB for CP, the mentioned constraints indicate the necessity for additional studies with stricter methodologies and bigger, more homogeneous groups to strengthen these results.

## 5 Conclusion

In conclusion, BFB shows significant promise as a supplementary treatment for different CP disorders. Across diverse populations, BFB demonstrated significant improvements in pain management, functional outcomes, and quality of life. While evidence strength varies, larger, well-designed randomized controlled trials could confirm its broader applicability. For LBP, surface EMG and RUSI-BFB showed consistent benefits in muscle activation and pain reduction. Similarly, BFB holds promise for headache, neck pain, fibromyalgia, and pelvic pain, although some studies noted limitations in study design and generalizability. For headaches and neck pain, BFB interventions not only alleviated discomfort but also improved muscle endurance, posture, and alignment, with some effects sustained over extended periods. Similarly, in conditions like fibromyalgia and pelvic pain, BFB contributed to symptom relief and better functional status. Future goals should prioritize carrying out bigger, well-organized randomized controlled trials to further solidify the long-term effectiveness of BFB techniques in managing pain. Moreover, investigating the best frequency and length of BFB treatments combined with the potential incorporation of BFB with other therapeutic approaches may provide valuable insights.

## CRediT authorship contribution statement

**Andrea Calderone:** Writing – original draft, Software, Resources, Investigation, Data curation, Conceptualization. **Vincenza Maura Mazzurco Masi:** Writing – original draft, Investigation, Formal analysis, Data curation. **Rosaria De Luca:** Visualization, Validation, Supervision, Project administration, Methodology, Investigation, Data curation, Conceptualization. **Antonio Gangemi:** Visualization, Validation, Supervision, Software, Resources, Investigation, Formal analysis, Data curation. **Mirjam Bonanno:** Visualization, Validation, Supervision. **Daniela Floridia:** Visualization, Validation, Supervision, Conceptualization. **Francesco Corallo:** Visualization, Validation, Supervision, Investigation, Conceptualization. **Giovanni Morone:** Visualization, Validation, Supervision. **Angelo Quartarone:** Visualization, Validation, Supervision, Funding acquisition. **Maria Grazia Maggio:** Writing – review & editing, Visualization, Validation, Supervision, Resources, Project administration, Methodology, Investigation, Data curation. **Rocco Salvatore Calabrò:** Writing – review & editing, Visualization, Validation, Supervision, Software, Resources, Project administration, Methodology, Funding acquisition, Conceptualization.

## Data availability statement

The data that support the findings of this study are not openly available due to reasons of sensitivity and are available from the corresponding author upon reasonable request.

## Funding

This study was supported by Current Research Funds 2024, 10.13039/501100003196Ministry of Health, Italy.

## Declaration of competing interest

The authors declare the following financial interests/personal relationships: Maria Grazia Maggio reports financial support was provided by 10.13039/501100003196Ministry of Health, Italy.
